# Seed priming with graphene oxide improves salinity tolerance and increases productivity of peanut through modulating multiple physiological processes

**DOI:** 10.1186/s12951-024-02832-7

**Published:** 2024-09-14

**Authors:** Ning Yan, Junfeng Cao, Jie Wang, Xiaoxia Zou, Xiaona Yu, Xiaojun Zhang, Tong Si

**Affiliations:** 1 Shandong Provincial Key Laboratory of Dryland Farming Technology, College of Agronomy, Qingdao Agricultural University, Qingdao, 266109 P.R. China; 2 School of Life Sciences, Centre for Cell & Developmental Biology and State Key Laboratory of Agrobiotechnology, The Chinese University of Hong Kong, Shatin, Hong Kong 999077 P.R. China

**Keywords:** Graphene oxide, Seed germination, Soil salinity, Production

## Abstract

Graphene oxide (GO), beyond its specialized industrial applications, is rapidly gaining prominence as a nanomaterial for modern agriculture. However, its specific effects on seed priming for salinity tolerance and yield formation in crops remain elusive. Under both pot-grown and field-grown conditions, this study combined physiological indices with transcriptomics and metabolomics to investigate how GO affects seed germination, seedling salinity tolerance, and peanut pod yield. Peanut seeds were firstly treated with 400 mg L⁻¹ GO (termed *GO priming*). At seed germination stage, GO-primed seeds exhibited higher germination rate and percentage of seeds with radicals breaking through the testa. Meanwhile, omics analyses revealed significant enrichment in pathways associated with carbon and nitrogen metabolisms in GO-primed seeds. At seedling stage, *GO priming* contributed to strengthening plant growth, enhancing photosynthesis, maintaining the integrity of plasma membrane, and promoting the nutrient accumulation in peanut seedlings under 200 mM NaCl stress. Moreover, *GO priming* increased the activities of antioxidant enzymes, along with reduced the accumulation of reactive oxygen species (ROS) in response to salinity stress. Furthermore, the differentially expressed genes (DEGs) and differentially accumulated metabolites (DAMs) of peanut seedlings under *GO priming* were mainly related to photosynthesis, phytohormones, antioxidant system, and carbon and nitrogen metabolisms in response to soil salinity. At maturity, *GO priming* showed an average increase in peanut pod yield by 12.91% compared with non-primed control. Collectively, our findings demonstrated that GO plays distinguish roles in enhancing seed germination, mitigating salinity stress, and boosting pod yield in peanut plants via modulating multiple physiological processes.

**Supplementary Information:**

The online version contains supplementary material available at 10.1186/s12951-024-02832-7.

## Introduction

High salt concentration is a significant constraint to crop growth, severely reducing productivity, especially under the ongoing global climate change scenario [1, 2]. To survive under salinity conditions, plants have evolved intricate regulatory mechanisms to minimize salt toxicity [3, 4]. From the perspective of plant growth and development, some plant species enhance their growth by promoting the assimilation, transportation, and distribution of nutrients like nitrogen (N), phosphorus (P), and potassium (K) to counteract high soil salinity [5, 6]. In some cases; however, the plant growth is inhibited under high soil salinity conditions for the purpose of stimulating plant salinity responses like activating cell signaling pathways, enhancing photosynthesis, and modulating antioxidant systems [4, 7]. The contradictions among the above results have attracted considerable attention, thus prompted us to further elucidate the coordination in the trade-off between plant growth and crop salinity resistance.

Legumes have long been recognized as important sources of proteins for human beings and livestock [8–10]. Legumes contribute significantly to sustainable agriculture and global food security by uniquely fixing atmospheric nitrogen through rhizobia symbiosis in their root nodules [11, 13]. As a typical representative of legumes, peanut is originated from South America, and is cultivated in arid and semi-arid areas worldwide [14, 15]. Compared with other oilseed crops, peanut seeds rich in unsaturated fatty acids (e.g. oleic acid, linoleic acid, and linolenic acid), which are beneficial to the cardiovascular protection of humans [16, 17]. In the past decade; however, soil salinity imposes severe limitations to peanut root growth, nodule development, N fixation capacity, and finally productivity [18–20]. Therefore, more eco-friendly and economical management practices are warranted to restrict the adverse impact of soil salinity on peanut production.

Graphene oxide (GO) is classified as a member of nanomaterials (NMs) family with multiple functions like adsorption, oxidation, and catalytic activity [21, 22]. In the practice of environmental science, GO has been prominently utilized for removal of heavy metals or organic pollutants in both contaminated soil and wastewater due to the properties of large pore volume and rich surface chemistry [86, 24]. In the past decade, the extensively utilization of GO in agricultural production has broaden our horizon in dissecting the prominent roles of NMs on crop science. To date, literatures have uncovered the profound role of GO application on plant abiotic stress responses which could be mainly ascribed to the following reasons: enhancement of plant growth via regulating nutrient assimilation [25, 26], protection of photosynthetic apparatus by facilitating electron transfer process [27, 28], and reduction of membrane lipid peroxidation through scavenging reactive oxygen species (ROS) [29, 30]. Nonetheless, obstacles still exist to utilize GO in crop production due to its versatility. It should be noted that the over-accumulation of GO in the soil could aggravate the toxicity of toxicants and pollutants, and finally interference with plant growth [31–33]. Seed priming is an eco-friendly and remarkable management strategy widely adopted by agronomists and farmers to confer soil salinity [34, 35]. Priming substances could stimulate the physiological and signalling processes of the sprouting seeds and invoke the plant salt tolerance in late growth stages without contaminating soil [36–38]; however, the mechanisms of NMs in seed priming are rarely known. Therefore, a research gap whether NMs like GO could be taken as a potential seed priming candidate in response to crop salinity stress should be properly addressed.

To favor our understanding of GO in peanut seed germination, salinity responses and pod productivity, the current study was carried out to test the hypothesis that seed priming with GO induced salinity tolerance in peanut seedlings is associated with the enhancement of plant growth, with a particular focus on the modulation of photosystem, antioxidant system, phytohormones, and carbon and nitrogen metabolisms. To this end, our study explored significant evidence from physiological, transcriptional, and metabonomic investigations using both pod-grown and field-grown experiments. The outcome of this study could provide a general guidance for the utilization of NMs to strengthen salinity resistance and increase productivity of legumes in the context of sustainable agriculture.

## Materials and methods

### Plant materials and treatments

#### Experiment I

**Fig. 1:**
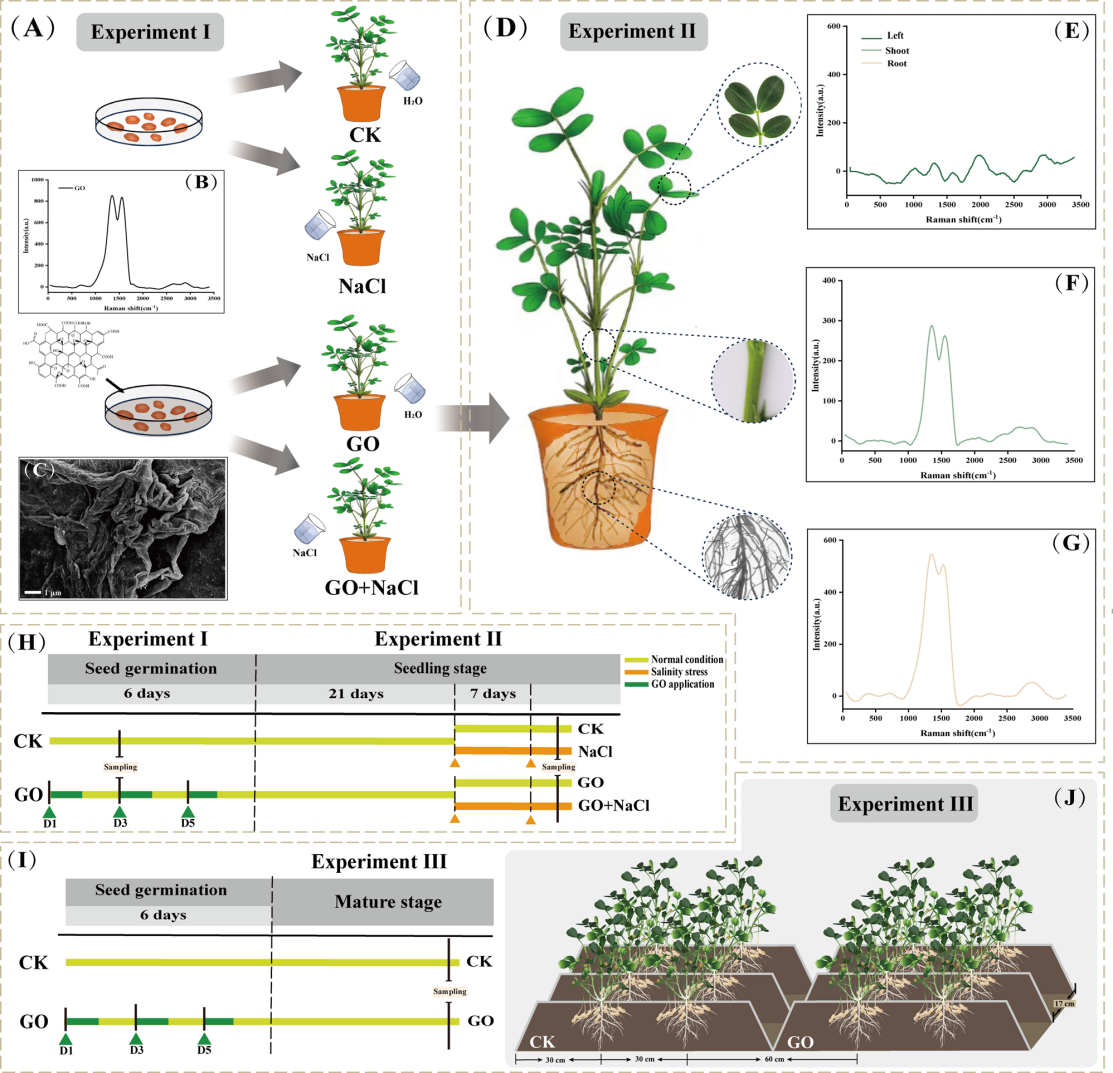
Schematic diagram (**H**) illustrating the experimental design and effects of seed priming with GO on promoting seed germination (**A**), alleviating seedling salinity stress (**D**), and enhancing productivity (**I & J**) of peanut. Characterization of GO in peanut seeds using SEM image (**C**). Characterization of GO in peanut seeds (**B**), leaves (**E**), stems (**F**), and roots (**G**) using Raman spectrum

To test the priming effects of graphene oxide (GO) on peanut (*Arachis hypogaea* L.) seed germination, seeds of the peanut cv. Huayu 25, a prominent cultivated variety of Shandong Province, were germinated in petri dishes. The investigation was performed at Qingdao Agricultural University, Qingdao, Shandong Province, China from January to May, 2023. Prior to germination, visually similar seeds underwent surface disinfection using a 1% sodium hypochlorite solution for 15 min, followed by thorough rinsing in sterile distilled water. Subsequently, half of the seeds were immersed in distilled water (termed *CK*), while the remaining seeds were exposed to 400 mg L^− 1^ graphene oxide (termed *GO priming*) in a dark environment at 28 °C. The GO was purchased from Daojin Technology Co. Ltd. (Beijing, China). In our preliminary experiment, 400 mg L^− 1^ has been proven to be the best concentration of graphene solution in stimulating peanut seed germination (unpublished data). After 24 h, the seeds were transferred to petri dishes with filter paper underneath (40 to 50 seeds in one dish). For “*CK*”, the seeds were applied with 50 mL of distilled water daily for 5 consecutive days. For “*GO priming*”, the seeds were treated with 50 mL of distilled water/400 mg L^− 1^ graphene solution daily at 2, 4, 6/3, 5 days after germination, respectively (Fig. 1A & H). The germination rate (GR) and percentage of seeds with radicals breaking through testa (PSWRB) were monitored for a period of 1 to 6 days. At 4 days after germination, seeds from both treatments were snap-frozen in liquid nitrogen and delivered to BioTree Biotechnology Co., Ltd. (Shanghai, China) for transcriptomic and metabolomics analysis.

#### Experiment II

To investigate the effects of *GO priming* on salinity tolerance of peanut seedlings, a pot-grown assay was conducted in Qingdao Agricultural University, Shandong Province, China from May to October, 2023. Seeds from *Experiment I*, namely “CK” and “GO” were sown in polystyrene pots (inner diameter of 9 cm and depth of 8 cm) separately. The seeds were sown 2 cm below the soil surface, with one seed per pot. Each pot contained 200 g of soil that had been heat-sterilized twice. The soil’s key properties included a pH of 6.83, bulk density of 1.19 g cm^−^³, organic matter content of 23.5 g kg^− 1^, total nitrogen at 90.2 mg kg^− 1^, available phosphorus at 28.3 mg kg^− 1^, and available potassium at 67.9 mg kg^− 1^. The pots were then moved to a greenhouse with the following conditions: a 16/8-hour photoperiod (light/dark), photosynthetic photon flux density (PPFD) of 1,000 µmol m⁻² s⁻¹, daytime air temperature of 25 °C, nighttime air temperature of 18 °C, and relative humidity of 75%. Each pot received 100 mL of distilled water every 2 days. 21 days after sowing, half of the “CK” and “GO” seedlings were subjected to salinity treatment by replacing distilled water with 200 mM NaCl solution in each pot at 22 and 29 days after sowing (Fig. 1D and H). Notably, the concentration of NaCl solution utilized in the current experiment was based on our preliminary experiments and previous reports [39–41]. In total, four treatments were composed: “CK”, “GO”, “NaCl”, and “GO + NaCl”. At 35 days after sowing, the seedlings were collected for the determination of agronomic characters and physiological parameters. Meanwhile, root samples from the four treatments were simultaneously snap-frozen in liquid nitrogen and delivered to BioTree Biotechnology Co., Ltd. (Shanghai, China) for transcriptomic and metabolomics analysis.

#### Experiment III

To investigate the effects of *GO priming* on peanut production, a field-grown experiment was carried out at Laixi experimental station, Shandong Province, China from May to October in 2022 and 2023. The chemical properties of the soil were determined before the initiation of the experiment (Table. S1). Basal synthetic fertilizer (750 kg ha^−1 ^; N: P_2_O_5_: K_2_O = 1: 1.5: 1.5) were applied before sowing. The field was cultivated with cereal and legume crops for 8 consecutive years. Peanut seeds treated as described in *Experiment I*, namely “CK” and “GO”, were sown in one seedling hole on 8 May and 9 May, in 2022 and 2023, respectively. The seeds were sown on a raised bed with two rows with bed height of 12 cm, bed width of 90 cm, row space of 30 cm, and hole space of 17 cm (Fig. 1I & J). Other agronomic practices such as irrigation, pesticide spraying, and weed control were carried out based on local farmers’ practices. The peanut pods were manually harvested on 13 September and 11 September, in 2022 and 2023, respectively. A total of three replicates (blocks) were composed with area of 45.0 m^2^ (4.5 m × 10.0 m) each. After sun-drying thoroughly, peanut pod yield as well as yield related components including plant number per hectare, pod number per plant, and 100-pod weight were measured.

### Synthesis of graphene oxide, scanning electron microscopy (SEM) and Raman Spectra analysis

A modified hummer’s method was utilized for the preparation of GO [42, 43]. In brief, 1 g of graphite power was added into a solution (130 mL concentrated sulfuric acid, 30 mL phosphoric acid, containing 6 g of KMnO_4_) and mixed at 0 °C. Then, the mixture was incubated in an oil bath at 50ºC for 12 h with continually stirring. After cooling, the solution was poured into a beaker containing 15 mL H_2_O_2_ and 100 mL distilled water under mechanical stirring until the solution turned into light yellow. Then, the solution was further stirred for 2 h to ensure the complete oxidation of graphite. Afterwards, the obtained turbid was centrifuged at 2000 *rpm* for 5 min until the black particles were separated. The turbid in the top of tube was washed with concentrated HCl, distilled water, and ethanol for three times, respectively. Finally, the products were freeze dried overnight to obtain the GO the with purity > 99wt%; oxygen content of 30–40%; layers of 1–5, thickness of 0.55 to 1.2 nm, and diameter ranging from and 0.5 to 3 μm.

The morphology and texture of GO in primed peanut seeds was characterized with a scanning electron microscope (SEM, JSM-7500 F, JEOL, Japan) following the method outlined by Cao et al. [21]. The plant samples (root, stem, and leaf) were washed twice with distilled water, dried, and ground into powder. Then the Raman spectrum of GO and the above samples were identified using Raman spectroscopy (DXR2xi, Thermo, USA) with a 532-nm excitation laser according to the protocol of Liu et al. [44].

### Plant morphology

Plant height was assessed by measuring the length of the main stem (from the cotyledonary node to the apical meristem). Subsequently, seedlings were categorized into different organs, and their fresh weight (FW) was recorded. The samples were then oven-dried at 105 °C for 30 min and stove-heated at 70 °C for 5 days to determine the dry weight (DW).

The peanut root morphology was detected based on our preliminary studies [41]. The root samples were completely dissected and thoroughly washed with distilled water. Then, the samples were scanned with a root scanning equipment (V700; SEIKO EPSON CORP.). The obtained data was further analyzed using WinRHIZO software (version 2013e; Regent Instruments Inc.).

### Transmission electron microscope (TEM) observation

The subcellular structure of peanut leaves was visualized using a cytochemical staining method [45, 46]. The freshly excised leaf tissue pieces (1–2 mm^2^) were firstly incubated in 1.25% (v/v) glutaraldehyde buffer at 4℃ for 4 h, and then in paraformaldehyde buffer (50 mM sodium cacodylate, pH 6.9) for another 4 h. Then the tissues were polymerized at 60℃ for 48 h after dehydrating in a graded ethanol series (30–100%; v/v). A Reichert-Ultracut E ultramicrotome was utilized to cut the leaf sections to 70–90 nm. Ultimately, the leaf sections were visualized at an accelerating voltage of 75 kV with a transmission electron microscope (HT7700; Hitachi, Tokyo, Japan).

### Gas exchange parameters

Gas exchange parameters were assessed on the third fully developed leaf of the main stem (termed functional leaf) in each seedling using established methods [39, 47] with a portable photosynthesis gas analyzer-coupled portable photosynthesis system (LI-6800, LI-COR, Lincoln, NE, USA) between 9:00 and 11:00 a.m. Measurements included net photosynthetic rate (Pn), stomatal conductance (Gs), transpiration rate (Tr), and intercellular CO_2_ concentration (Ci). The conditions in the leaf chamber (2 cm × 3 cm) were set to an ambient CO_2_ concentration of 400 µmol mol^− 1^, a PPFD of 1,000 µmol m^− 2^ s^− l^, an air temperature of 25 ± 1℃, and a relative air humidity of 80%.

### Chlorophyll fluorescence and chlorophyll content

The chlorophyll fluorescence parameters of the functional leaf were determined using a chlorophyll fluorescence imaging system (Imag-Maxi, Heinz Walz, Effeltrich, Germany) based on our preliminary experiments [40, 48]. After the treated seedlings were dark adapted for 30 min, the third fully developed leaf of the main stem was cut down to analysis the chlorophyll fluorescence parameters such as the maximal photochemical efficiency of Photosystem II (PSII) (Fv/Fm), quantum yield of PSII photochemistry (ΦPSII), and photochemical quenching coefficient (qP) as area of interest. The chlorophyll fluorescence parameters were calculated by the FluorImager software (Version 2.2; Technologica Ltd., United Kingdom).

To determine the total chlorophyll content, 0.05 g leaves were accurately weighed and transferred to 25 mL glass scale tubes, and then extracted by adding 25 mL mixture of acetone and ethanol (1:1, v/v). Then, the samples were incubated for 12 h under dark at 40℃ and mixed thoroughly for several times. The absorbance values at 663 and 645 nm were measured by an UV-Vis spectrophotometer (Cary 60, Agilent, USA). The total chlorophyll content was calculated as described by Lichtenthaler and Wellburn [59] using the following formula:

Ca = 12.72×A663-2.59×A645 (1).

Cb = 22.88×A645-4.67×A663 (2).

Ct = Ca + Cb = 20.29×A645 + 8.05×A663 (3).

where Ca is chlorophyll a content (mg·L^− 1^), Cb is chlorophyll b content (mg·L^− 1^), Ct is total chlorophyll content (mg·L^− 1^), and A663 and A645 represent absorbance at 663 and 645 nm, respectively.

### Relative water content and relative electrolyte conductivity

The relative water content (RWC) of the functional leaf was measured according to Jensen et al. [50]. The excised leaves were soaked in 10 mL of distilled water for 4 h under dark at 4℃ to obtain the turgid weight (TW). The fresh weight (FW) and dry weight (DW) were determined as mentioned above. RWC was calculated as the following formula: RWC (%) = [(FW − DW) / (TW − DW)] × 100.

The relative electrolyte conductivity (REC) was analyzed with a conductivity bridge (DDS-307 A, LEX Instruments Co., Ltd., China) following the protocol of Griffith and Mclntyre [51]. The functional leaves were soaked in 10 mL of distilled water for 12 h under dark at 25℃ to obtain the conductivity (C1). After which the solution was boiled for 30 min and the conductivity (C2) was measured again after cooling. REC (%) was calculated as: C1/C2 × 100%.

### Histochemical staining and quantitative assay of H_2_O_2_ and O_2_^−^

Hydrogen peroxide (H_2_O_2_) within the functional leaf was visually detected in situ through the application of 3,3’-diaminobenzidine (DAB) staining [52]. Leaf samples were submerged in a DAB solution at a concentration of 1 mg mL^− 1^ (pH 3.8) and incubated for 12 h at 25℃, under a PPFD of 1000 µmol m^− 2^ s^− 1^. Subsequently, the leaves underwent bleaching in boiling ethanol at a concentration of 95% (v/v) and were then immersed in a lactic acid/phenol/water mixture at equal parts (1:1:1, v/v/v) for imaging purposes. Similarly, the in situ visual detection of superoxide anion (O_2_^−^^.^) was performed using nitro blue tetrazolium (NBT) staining [53]. The leaf samples were immersed in a 1 mg mL^− 1^ NBT solution (pH 6.1) for 8 h at 25℃ in the absence of light. The bleaching and imaging process for these samples was identical to that described above.

For the quantitative assay of hydrogen peroxide (H_2_O_2_), 0.2 g fresh functional leaf samples were excised and homogenized immediately with pre-cooled 2 mL of 0.2 M HClO_4_. After centrifuging at 6,000 *g* for 5 min at 4℃, the supernatant was collected and adjusted to pH 6.5. After centrifuging at 12,000 *g* for 5 min at 4℃, H_2_O_2_ was eluted from the supernatant and mixed with 0.4 mL reaction buffer containing 4 mM 2, 2’-azino-di-(3-ethylbenzthiazoline-6-sulfonic acid), 100 mM potassium acetate, and horseradish peroxidase (0.5 U). Then, the quantification of H_2_O_2_ levels was ascertained by monitoring the absorbance shift of the titanium peroxide complex at a wavelength of 412 nm [55].

For the quantitative assay of O_2_^−·^ production rate, 0.2 g fresh functional leaf samples were excised and homogenized immediately with 2 mL of pre-cooled phosphate buffer (50 mM, pH 7.8). After centrifuging at 12,000 *g* for 20 min at 4℃, the supernatant was collected for the subsequent determination. Then, 0.5 mL of the supernatant was mixed thoroughly with 0.5 mL of phosphate buffer (50 mM, pH 7.8) and 1 mL of hydroxylamine hydrochloride (10 mM). After incubating for 1 h at 25℃, 1 mL of p-aminobenzene sulfonic acid (17 mM) and 1 mL of α-naphthylamine (7 mM) were added and incubated for another 20 min at 25℃. After centrifuging at 3,000 *g* for 5 min, the O_2_^−·^ production rate was quantified by monitoring the synthesis of nitrite at 530 nm [27].

### Antioxidant enzyme activities and malondialdehyde content

Fresh samples weighing 0.5 g from the functional leaves were pulverized using a pre-cooled mortar and pestle, mixed with 5 mL of ice-cold phosphate buffer (50 mM, pH 7.8) that included 20% glycerol (v/v), 0.2 mM EDTA, 5 mM MgCl_2_, 1 mM AsA, 1 mM GSH, and 1 mM DTT. This mixture was then subjected to centrifugation at 12,000 *g* for 20 min at 4℃. The resulting supernatant was utilized to evaluate the activity of various antioxidant enzymes, namely superoxide dismutase (SOD), catalase (CAT), guaiacol peroxidase (G-POD), ascorbate peroxidase (APX), and to measure the levels of malondialdehyde (MDA) equivalents. The activity of SOD was measured at 560 nm, reflecting its capacity to inhibit the photochemical reduction of nitro blue tetrazolium (NBT), according to the method of Stewart and Bewley [56]. CAT activity was gauged by the rate of H_2_O_2_ degradation, observed at 240 nm, adapting the method by Patra et al. [57]. G-POD activity was assessed with a guaiacol substrate at 470 nm based on the procedure of Cakmak and Marschner [58]. APX activity was determined through the rate of ascorbate oxidation at 290 nm based on the protocol of Nakano and Asada [59]. The content of MDA was ascertained using the thiobarbituric acid (TBA) reaction method, with absorbance readings of the red adduct taken at 450, 532, and 600 nm to compute the MDA equivalents [60].

### Accumulations of total soluble sugar, sucrose, and free amino acids

The dry samples of peanut roots were firstly powdered by a high-speed ball mill (MM400; Retsch GmbH, Haan, Germany). Then, 0.1 g of the samples was extracted with 8 mL of 80% (v/v) ethanol at 80℃. After centrifugation at 3000 *g* for 30 min, the supernatant was collected for the subsequent determination. The total soluble sugar content was analyzed at 620 nm based on the anthrone method [61]. The sucrose content was determined at 480 nm following the resorcinol method as described by Buysse and Merckx [61]. The free amino acids (FAA) content was evaluated at 570 nm following the ninhydrin reaction as modified by Moore and Stein [62].

### Contents of nitrogen, phosphorus, and potassium

The dry samples of peanut roots were powered as mentioned above and digested with H_2_SO_4_-H_2_O_2_. The total nitrogen (N) content was determined using the micro Kjeldahl method [63]. The total phosphorus (P) content was measured by a continuous flow analyzer based on the procedure of Khashi u Rahman et al. [64] with minor modifications. The total potassium (K) content was determined by using a flame photometer following the method of Chakraborty et al. [65].

### Total RNA extraction and RNA-seq analysis

Freshly excised peanut root samples were used for total RNA extraction using the Cetyltrimethylammonium bromide (CTAB) method [66]. The RNA’s purity, concentration, and integrity were assessed with a NanoPhotometer^®^ spectrophotometer (IMPLEN, CA, USA), a Qubit^®^ RNA Assay Kit in a Qubit^®^ 2.0 Fluorometer (Life Technologies, CA, USA), and an RNA Nano 6000 Assay Kit on the Bioanalyzer 2100 system (Agilent Technologies, CA, USA), respectively [67]. Subsequently, 1 µg of high-quality RNA per sample was used to construct the cDNA library, which was sequenced on an Illumina HiSeq platform by Novogene Corporation Inc. The software fastp v0.19.3 was employed to clean and trim the raw data, ensuring high-quality clean reads by filtering out reads with adapters, paired reads with over 10% N content, and reads where more than 50% of the bases had a quality score of Q ≤ 20 [68]. The clean data were then mapped to the peanut reference genome (https://peanutbase.org/) using HISAT v2.1.0 software. The expression abundance of reads was quantified using the fragments per kilobase of transcript per million base pairs (FPKM) value. Differentially expressed genes (DEGs) between the groups were identified using DESeq2 v1.22.1 (Ross Ihaka, University of Auckland, New Zealand) with a threshold of |log_2_ Fold Change| ≥ 1 and a False Discovery Rate (FDR) < 0.05 [69]. Gene Ontology (GO) enrichment analysis and the Kyoto Encyclopedia of Genes and Genomes (KEGG) pathway annotation (http://www.genome.jp/kegg/) of DEGs were performed as reported [70].

### Quantitative real-time PCR

The identical total RNA specimens from the aforementioned RNA-seq study were employed in the subsequent quantitative real-time PCR (qRT-PCR) evaluation [71]. Details regarding the specific primers are presented in Table S2. For the synthesis of cDNA, 1 µg of total RNA was utilized, employing the TransScript^®^ II First-Strand cDNA Synthesis SuperMix (AH301-03, TransGen Biotech Co., Ltd, Beijing, China) [72]. The products, once diluted to a tenfold degree, were then applied to the quantitative real-time PCR (qRT-PCR) process [73], which was conducted using SYBR^®^ Green Pro Taq HS (AG11701, Accurate Biotechnology (Hunan) Co., Ltd, Changsha, China) on the ECO real-time PCR system by Illumina [74].

### Non-targeted metabolites extraction and determination

To identify the root-specific metabolites, non-targeted metabolite profiling was conducted. Briefly, root samples were meticulously cleansed with distilled water, then snap-frozen in liquid nitrogen, and dispatched to Gene Denovo Biotechnology Co., Ltd. for metabonomic evaluation [75, 76]. The specimens underwent LC-MS/MS analysis using a Vanquish UHPLC system (Thermo Fisher Scientific). Subsequently, the raw data were transformed into mzXML format. High-resolution mass spectrometry data were then processed using MAPS and matched against an MS2 database for identification. Hierarchical clustering analysis followed, generating a dendrogram via the average linkage method, as detailed by Yuan et al. [77]. Principal component analysis (PCA) was also executed to delineate group distinctions. The (O)PLS model’s variable importance in projection (VIP) scores were employed to prioritize the differentially accumulated metabolites (DAMs) that most effectively differentiated the groups. Metabolites exhibiting a T-test *P*-*value* < 0.05 and a VIP score ≥ 1 were deemed significantly different between the groups. Conclusively, KEGG pathway enrichment analysis was performed for metabolite annotation [78, 79]. Both positive and negative ion modes were integrated for the entire analysis. *Experiment I* comprised three replicates while *Experiment II* included four.

### Statistical analysis

The experimental design for the measurements employed a fully randomized model, incorporating three biological duplicates: each consisting of three petri dishes in *Experiment I* and ten seedlings in *Experiment II*, excluding metabonomic assessments. Results were presented as mean values ± standard deviation (SD) and subjected to one-way and multi-factor ANOVA using SPSS 22.0 (SPSS Inc.). The disparities between treatments were assessed for significance using Tukey’s test.

## Results

### Characteristics of graphene oxide in peanut seeds and seedlings

The GO used in the current study was firstly detected with a SEM. In *Experiment I*, the GO in the primed seeds exhibited a stacked and folded form with layered structure (Fig. 1C). Raman spectroscopy further showed the representative D and G peaks of GO in primed seeds (Fig. 1B). In *Experiment II*, the representative D and G peaks of GO were only observed in peanut stems and roots (Fig. 1F and G), other than leaves (Fig. 1E) in GO-primed seedlings. These observations indicate that seed priming is an effective way to accumulate GO during the peanut seedling stage.

### Graphene oxide promoted the germination of peanut seeds

**Fig. 2:**
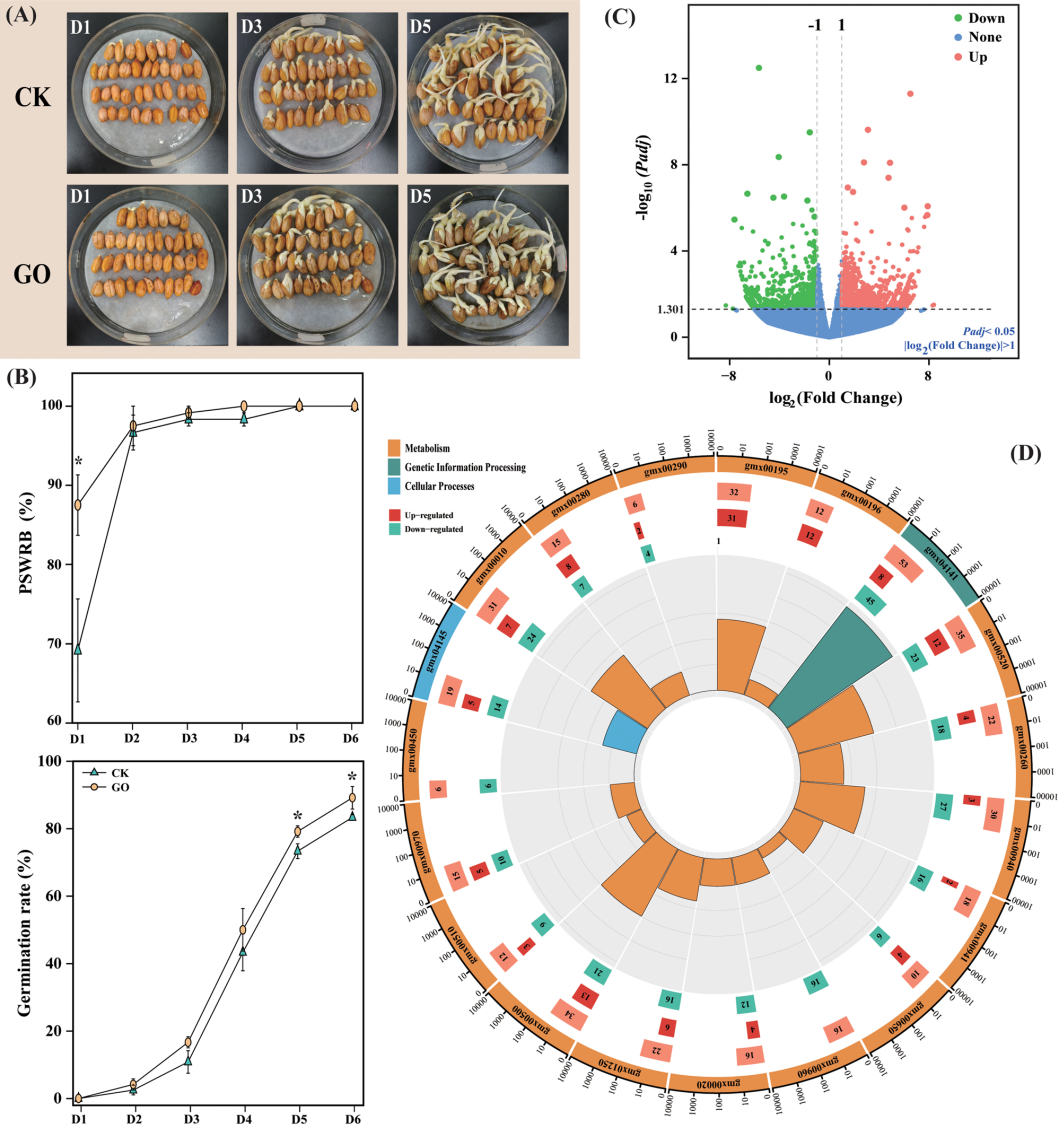
Effects of seed priming with GO on phenotypes of peanut seeds (**A**), percentage of seeds with radicals breaking through testa (PSWRB) and germination rate (**B**) during germination. (**C**) Volcano plot of the DEGs in GO-treated peanut seeds. (**D**) KEGG pathway analyses of the enriched DEGs in GO-treated peanut seeds. The enrichment circle diagram is from outside to inside, and the first circle is the KEGG pathway ID label. The strip length of the second circle corresponds to the enriched DEGs of the pathway, which represents the number of total DEGs, up-regulated DEGs and down-regulated DEGs, respectively. The third circle (polar histogram) is Rich factor

To examine the effect of *GO priming* on seed germination of peanut, we conducted *Experiment I*. Seed priming with GO significantly increased the PSWRB by 25.51% compared with CK at 1 day after germination whereas no significant difference was observed along the rest time course of germination process. By contrast, GO-primed seeds exhibited a significant increase in germination rate by 7.95% and 7% at 5 and 6 days after germination, respectively, compared with the non-primed seeds (Fig. 2A & B). RNA-seq analysis was conducted to further elucidate the expression levels of the genes associated with GO. A total of 1703 DEGs (935 up-regulated and 768 down-regulated) were detected in GO treated peanut seeds compared with CK (Fig. 2C). qRT-PCR analysis indicated that the selected genes exhibited similar expression patterns with the RNA-seq data Fig. S5).

Based on the KEGG pathway enrichment analysis (Fig. 2D), the majority of the top 19 enriched pathways were related to metabolic processes. These include pathways such as “Starch and sucrose metabolism”, the “TCA (tricarboxylic acid) cycle” involved in sugar metabolism, and “Glycine, serine, and threonine metabolism” associated with protein metabolism. Additionally, secondary metabolic processes like “Flavonoid biosynthesis” and “Phenylpropanoid biosynthesis” were also enriched (Table. S3). Furthermore, according to KEGG and Gene Ontology enrichment analyses, intracellular information exchange and protein processing were also enriched (Figs. 2C and S1). Specifically, pathways related to protein processing in the “Endoplasmic Reticulum” and “Golgi-associated vesicles” showed enrichment. These findings suggest that *GO priming* may influence the seed primary metabolism (sugar and protein) as well as secondary metabolisms, thus potentially promotes seed germination.

### Graphene oxide promoted peanut seed growth by regulating amino acid and secondary metabolisms

**Fig. 3:**
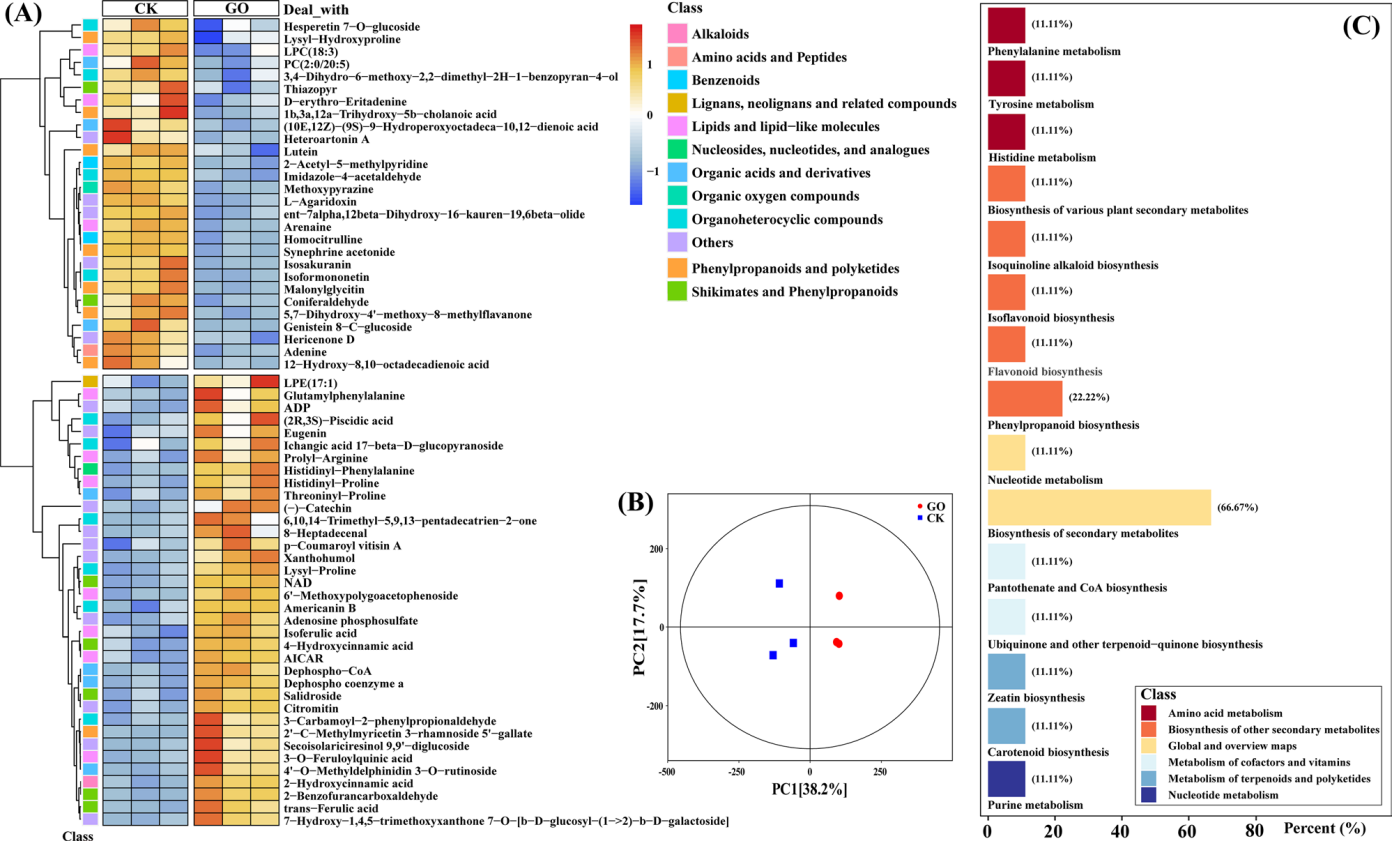
Dynamical changes of DAMs in GO-treated peanut seeds compared with CK. (**A**) Identified metabolites were clustered based on their abundance relative to control samples. The red and blue colors of the boxes represent upregulated and downregulated DAMs, respectively. (**B**) Principal Component Analysis (PCA) of metabolites in different groups. (**C**) KEGG classification of DAMs in response to GO

The non‑targeted metabolites assay was further carried out to elucidate the role of *GO priming* on seed metabolite profiles. PCA analysis indicated that the first and second principal components were displayed on the X (PC1, 38.2%) and Y (PC2, 17.7%) axis, respectively. The replicate samples clustered together and obvious separations have been observed between GO and CK (Fig. 3B). A total of 64 DAMs (36 up-regulated and 28 down-regulated) have been identified in GO-treated seeds compared with CK (Table. S4). Particularly, most of the DAMs could be mainly classified as “Alkaloids”, “Amino acids”, “Benzenoids”, and “Organic acids” (Fig. 3A). Furthermore, the differentially abundant metabolites were conducted using KEGG database. Pathways associated with “Amino acid metabolism” and “Secondary metabolites biosynthesis” exhibited higher degree of alteration in GO-primed peanut seeds compared with non-primed control (Fig. 3C).

### Graphene oxide enhanced peanut seedling growth by modifying nitrogen metabolism and phytohormones biosynthesis under salinity stress

**Fig. 4:**
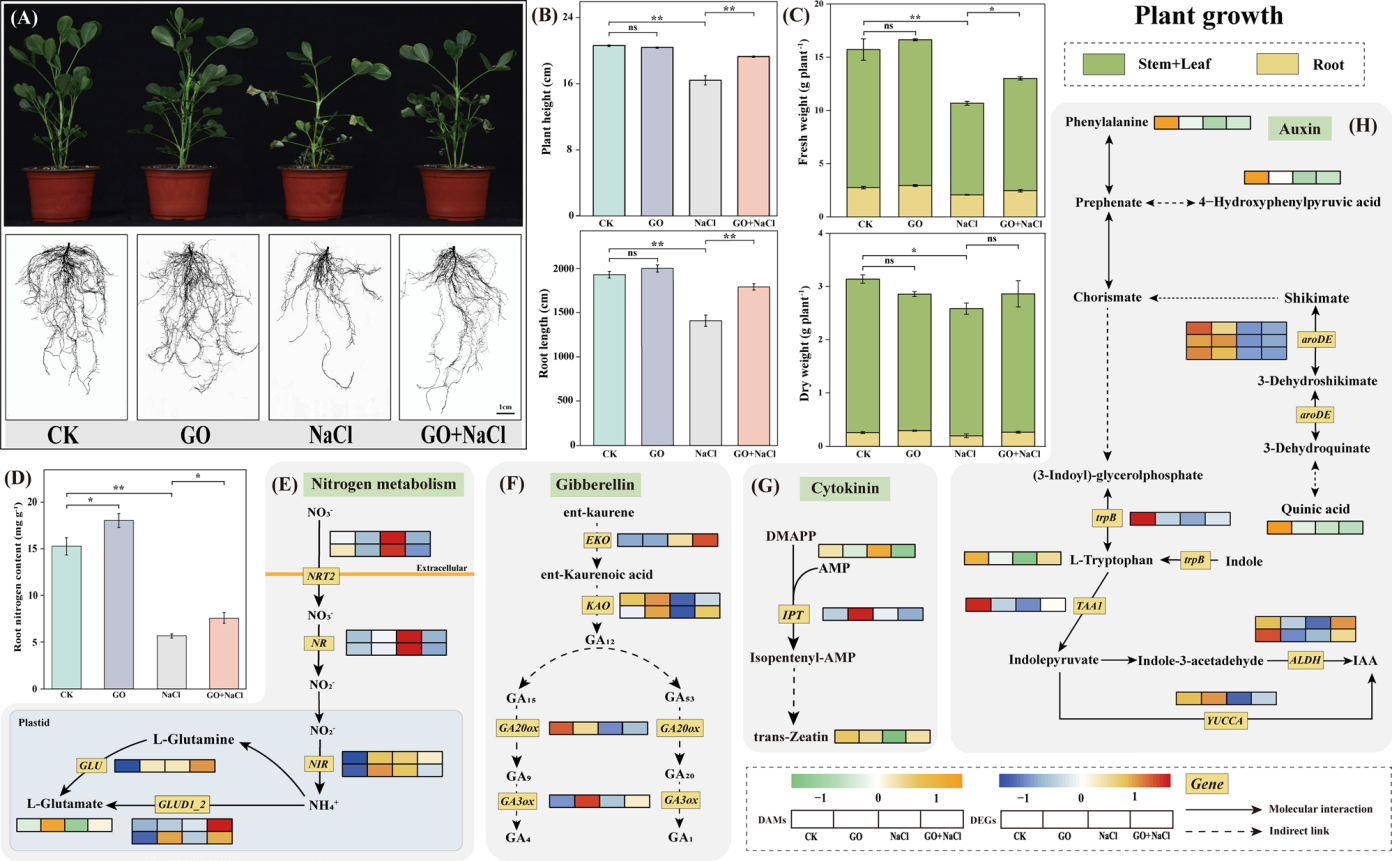
GO alleviates peanut salinity stress through enhancing plant growth. (**A**) Phenotype analysis of seedlings under different treatments. Statistical analysis of the aboveground and underground parts of peanut seedlings under different treatments. (**B**) Plant height and root length. (**C**) Fresh weight (FW) and dry weight (DW) of stem + leaf and root. (**D**) Total nitrogen content of roots. mean ± SD (*n* = 3), “ns” non-significant, *P<0.05, **P<0.01, Tukey’s test. Heatmap of DEGs and DAMs in nitrogen metabolism (**E**) and plant hormone metabolism including gibberellin (**F**), cytokinin (**G**) and auxin (**H**), respectively. Grids represent the expression levels of genes, which were shown as FPKM values, *Padj* < 0.05. The red and blue colors of the boxes represent up-regulated and down-regulated genes, respectively. The orange and green colors of the boxes represent up-regulated and down-regulated metabolites, respectively

To explore the impact of *GO priming* on the salinity stress tolerance of peanut seedlings, we conducted *Experiment II*. The results revealed that, compared to the control group, GO treatment alone had few effects on peanut growth and development (Fig. 4A, B and C). However, under salinity conditions, GO-treated peanut seedlings exhibited significant improvements in both above-ground and below-ground growth (Fig. 4A). Consequently, we focused on the effects of salinity stress (abbreviated as “NaCl”) in conjunction with *GO priming* (abbreviated as “GO + NaCl”). When compared to salinity stress alone, *GO priming* led to a remarkable increase of 17.47% in plant height, 27.22% in root length, and 26.28% in fresh weight, while the increase in dry weight was not statistically significant under salinity stress (Fig. 4B & C). Furthermore, we conducted transcriptome sequencing for each treatment, identifying 991 DEGs shared between “GO + NaCl vs. CK” and “NaCl vs. CK” Fig. S2A). KEGG enrichment analysis revealed their association with nitrogen utilization, secondary metabolism, and plant hormones Fig. S2B). Additionally, we performed metabolomic profiling, which showed similar patterns to the phenotypic results. PCA indicated that the “CK” and “GO” groups were more closely related, while “NaCl” and “GO + NaCl” exhibited distinct metabolic features at the metabolic level Fig. S3A). Further KEGG analysis of DAMs revealed significant enrichment in pathways related to proteins, sugars, and plant hormones Fig. S3B & C).

GO has been reported to enhance nutrient absorption in plants [21]. To investigate whether graphene affects nutrient uptake under salt stress, we measured the carbon, phosphorus, and potassium content in the roots. The results showed that *GO priming* significantly increased the nitrogen, phosphorus, and potassium content in the roots compared to salinity stress alone (Figs. 4D and S4). Specifically, nitrogen, phosphorus, and potassium levels increased by 44.37%, 88.62%, and 29.90%, respectively (Figs. 4D and S4). Notably, the *NRT2* transporter, responsible for nitrogen uptake, exhibited the highest expression under salt stress, likely due to feedback regulation caused by nutrient deficiency. Additionally, in the plastids, *GLU* and *GLUD1_2* expression was significantly higher in “GO + NaCl”, leading to the recovery of glutamate levels (Fig. 4E).

Regarding hormones, we analyzed gene expression and content related to growth hormones and stress-responsive hormones (Fig. 4F, G and H). Upstream rate-limiting enzymes in the gibberellin (GA) pathway, *KAO* and *GA20ox* were significantly reduced under salinity stress whereas *GO priming* partially restored their expression (Fig. 4F). GA1 and GA4, the biologically active forms of GA in plants, are catalyzed by *GA3ox*. While salt stress suppressed the expression of this gene, *GO priming* promoted the reaction by upregulating the expression of *GA3ox* (Fig. 4F), aligning with the observed phenotypic changes **(**Fig. 4A**)**. A similar pattern was observed for auxin: the precursor of IAA- L-tryptophan content was lowest under salinity stress whereas *GO priming* partially restored it. Furthermore, key enzymes involved in IAA synthesis, ALDH and YUCCA, exhibited higher expression in “GO + NaCl” compared with “NaCl” (Fig. 4H). In terms of stress hormones, we found that the cytokinin precursor DMAPP accumulated under salt stress, while the downstream key enzyme *IPT* expression was weakened. Consequently, the final cytokinin content was lower in “NaCl” than in “GO + NaCl” (Fig. 4G). Overall, although standalone *GO priming* had limited effects on peanut seedling growth, it enhanced salinity stress tolerance by regulating nitrogen assimilation and plant hormone metabolism.

### Graphene oxide regulated osmoregulation and carbon metabolism of peanut seedlings in response to salinity stress

**Fig. 5:**
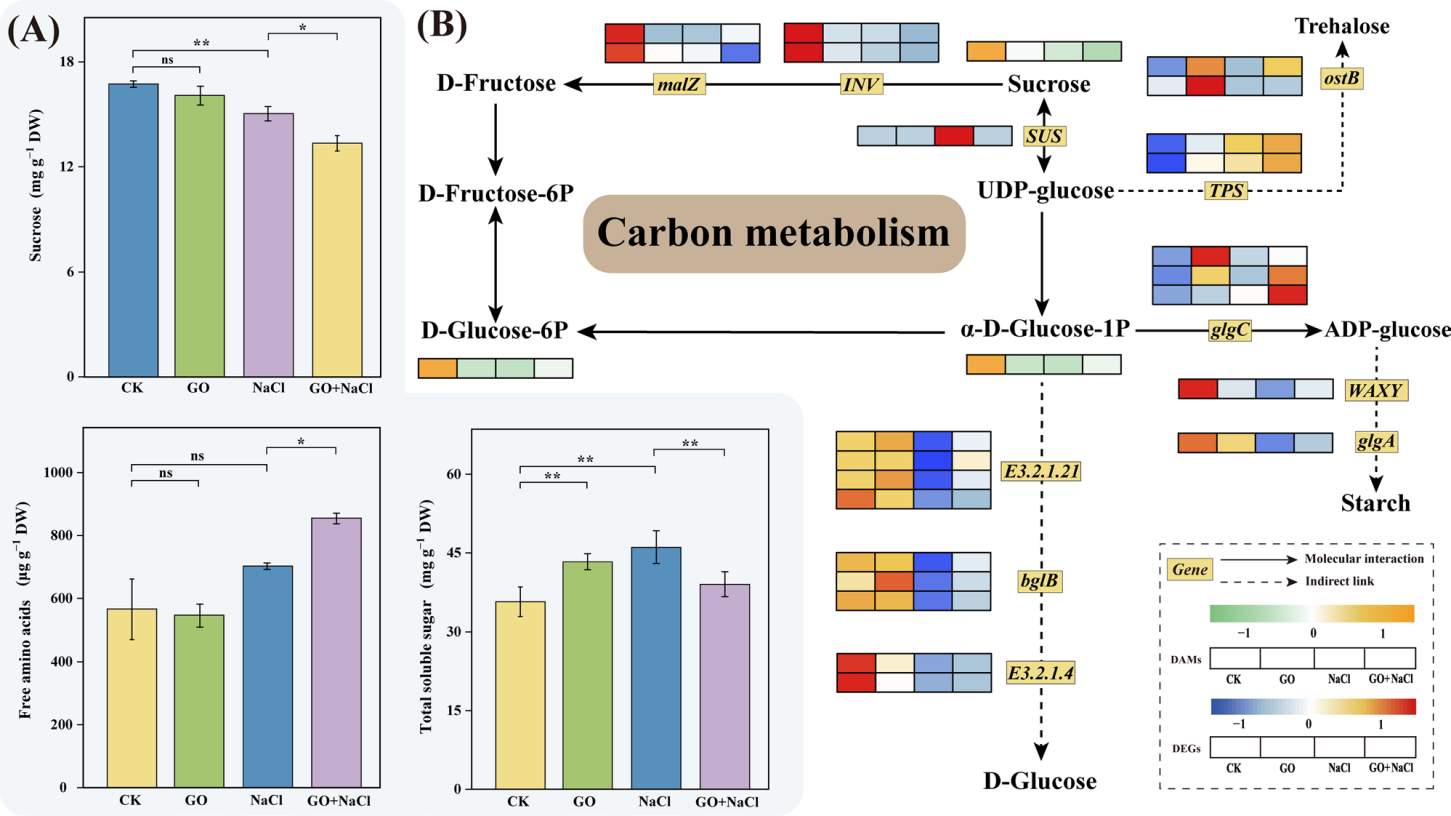
GO alleviates peanut salinity stress through modulating carbon metabolism. (**A**) Contents of sucrose, free amino acids, and total soluble sugar. mean ± SD (*n* = 3), “ns” non-significant, *P<0.05, **P<0.01, Tukey’s test. (**B**) Heatmap of DEGs and DAMs in carbon metabolic pathways. Grids represent the expression levels of genes, which were shown as FPKM values, *Padj* < 0.05. The red and blue colors of the boxes represent up-regulated and down-regulated genes, respectively. The orange and green colors of the boxes represent up-regulated and down-regulated metabolites, respectively

To further investigate the impact of *GO priming* on peanut salt tolerance, we conducted analyses on soluble sugars, free amino acids, and total soluble sugars in the plant roots (Fig. 5A). The results revealed that the combination of GO and NaCl led to lower levels of soluble sugars and sucrose compared to salt treatment alone. Interestingly, the content of free amino acids in the “GO + NaCl” was approximately 10% higher than in the “NaCl” (Fig. 5A). Given these findings, we speculate that *GO priming* helps to maintain the osmotic pressure of peanut roots under salt stress, thereby enhancing salt tolerance.

Furthermore, we observed that the expression levels of genes involved in D-fructose synthesis (*INV* and *malZ*) were lower in the GO + NaCl treatment compared to salt treatment alone (Fig. 5B). Conversely, genes responsible for D-glucose synthesis exhibited the opposite expression pattern. Additionally, genes (*TPS* and *ostB*) related to the synthesis of trehalose, which could protect the cells in high-osmotic environments, were upregulated in the “GO + NaCl” group compared with “NaCl”. On another note, the synthesis of non-soluble starch sugars also showed some improvement under GO supplementation compared to “NaCl” (Fig. 5B). Taken together, *GO priming* influences carbon metabolism and osmotic regulation in peanut under salinity stress.

### Graphene oxide increased the photosynthesis and strengthened the photosystem of peanut seedlings in resistance to salinity stress

**Fig. 6:**
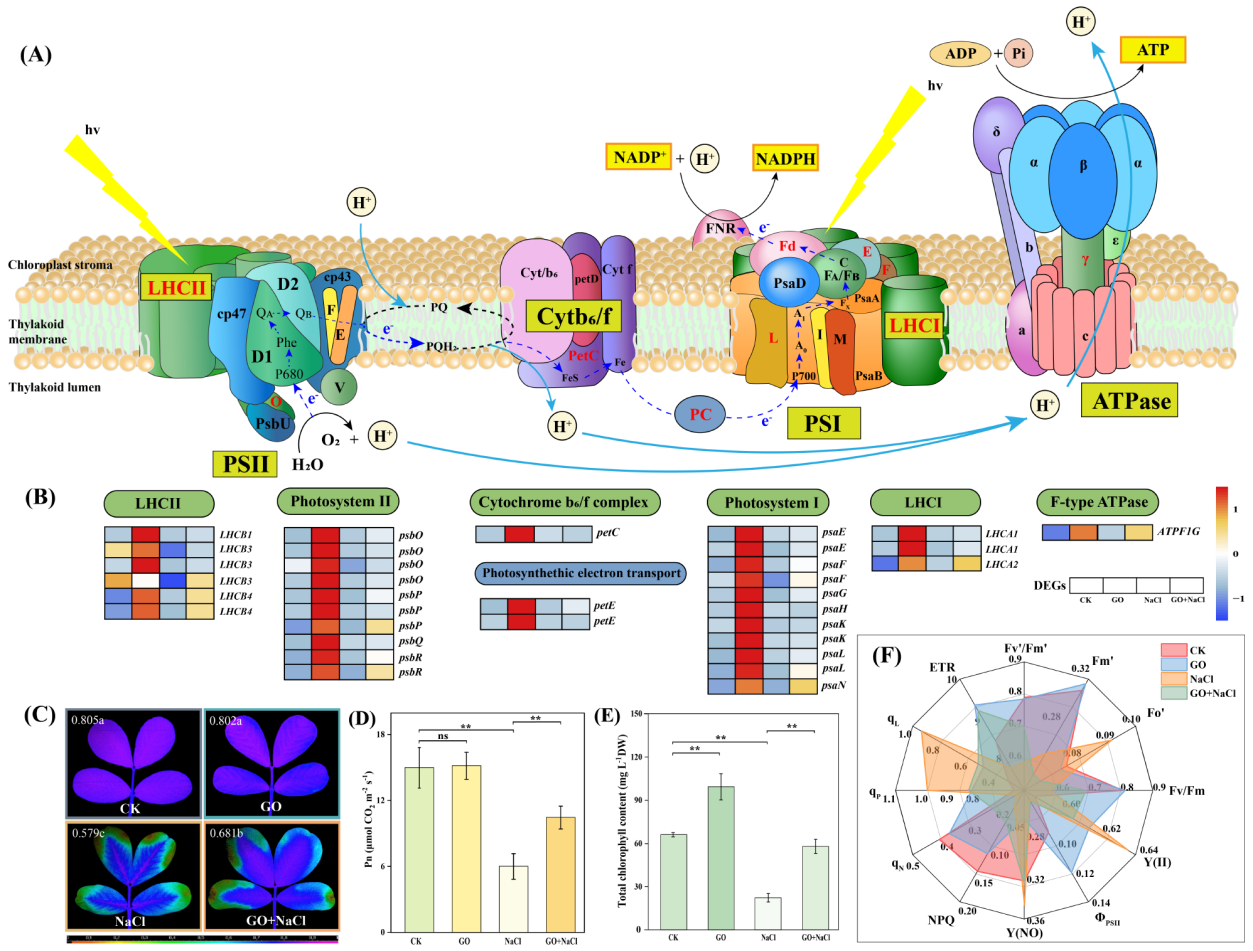
GO alleviates peanut salinity stress via regulating photosystem. (**A**) Schematic diagram of the photosynthetic system in plant photosynthesis. (**B**) The expression profile of transcripts involved in photosynthesis. Grids represent the expression levels of genes, which are shown as FPKM values, *Padj* < 0.05. The red and blue colors of the boxes represent up-regulated and down-regulated genes, respectively. (**C**) Maximal photochemical efficiency of photosystem II (PSII) (Fv/Fm). The false-color code, depicted at the bottom of the image, ranges from 0 (black) to 1 (purple). Different lowercase letters on imagines indicate significant differences among treatments. mean ± SD (*n* = 3), *P*<0.05, Tukey’s test. (**D**) Net photosynthetic rate (Pn). (**E**) Leaf total chlorophyll content. mean ± SD (*n* = 3), “ns” non-significant, *P<0.05, **P<0.01, Tukey’s test. (**F**) Radar maps of some crucial chlorophyll fluorescence parameters

We further investigated the role of *GO priming* in the regulation of peanut photosystem under salinity stress (Fig. 6A). Standalone *GO priming* showed little effects on Fv/Fm and Pn while significantly increased the total chlorophyll content by 50.14% under stress-free conditions. Soil salinity significantly reduced the Fv/Fm, Pn, and total chlorophyll content whereas *GO priming* significantly increased the Fv/Fm, Pn, and total chlorophyll content by 17.62, 74.15, and 158.23%, respectively, under salinity stress (Fig. 6C, D and E). Apart from Fv/Fm, some major chlorophyll fluorescence parameters including ΦPSII, Fv’/Fm’, and ETR were dramatically increased whereas NPQ was reduced by *GO priming* (Fig. 6F). RNA-seq analysis further revealed that *GO priming* significantly elevated the expression of some crucial genes regarding the components of photosynthetic chain (LHCII, Photosystem II, Cytochrome b_6_/f complex, Photosystem I, LHCI, and F-type ATPase) under both stress-free and salinity conditions (Fig. 6B). These results provide evidence that *GO priming*-induced alleviation effects of peanut salinity stress is associated with the strengthened photosystem.

### Graphene oxide enhanced the antioxidant system and maintained the plasma membrane integrity of peanut seedlings under soil salinity conditions

**Fig. 7:**
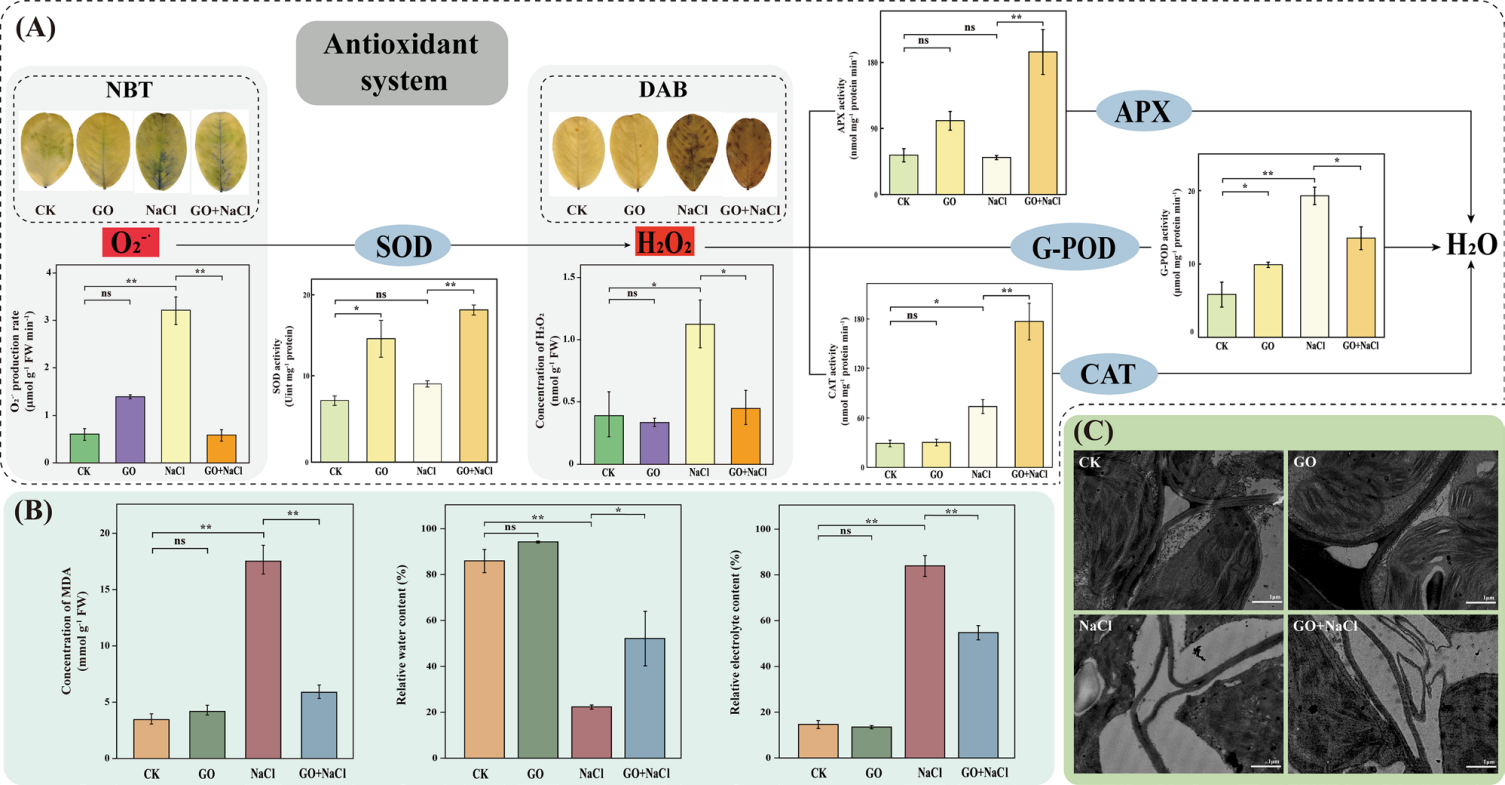
GO alleviates peanut salinity stress through maintaining antioxidant system and plasma membrane integrity. (**A**) Concentrations of O_2_^−^^.^ and H_2_O_2_, histochemical analysis of O_2_^−^^.^ and H_2_O_2_ by NBT and DAB staining, and activities of antioxidant enzymes (SOD, APX, G-POD, and CAT). (**B**) Concentrations of MDA, RWC, and REC. mean ± SD (*n* = 3), “ns” non-significant, *P<0.05, **P<0.01, Tukey’s test. (**C**) Observation of the subcellular structure of peanut leaves by TEM. Scale bar = 1 μm

To investigate the role of *GO priming* in the modulation of peanut antioxidant system under salinity stress, we firstly assessed the activities of crucial antioxidant enzymes. Standalone GO treatment significantly increased the activities of SOD (100.59%) and G-POD (69.61%) compared with CK. Strikingly, the activities of SOD, APX, and CAT were dramatically increased by 94.88, 289.19, and 139.89%, respectively, under salinity conditions in peanut leaves of *GO priming* (Fig. 7A). In accordance with the antioxidant enzyme data, the histochemical staining and quantity assay of ROS further indicated that *GO priming* significantly reduced the accumulation of H_2_O_2_ and O_2_^−^^.^ in peanut leaves under salinity stress (Fig. 7A). We further detected the cell membrane peroxidation through evaluating the synthesis of MDA where seedlings of *GO priming* exhibited lower concentration of MDA under soil salinity conditions (Fig. 7B). Under stress-free conditions, no significant difference was detected between *GO priming* and CK plants in RWC and REC. Under salinity growth conditions, *GO priming* significantly increased the leaf RWC by 134.08% while significantly reduced the leaf REC by 53.35%, compared with control (Fig. 7B). Moreover, no significant difference was observed between “GO” and “CK” in the subcellular structure of peanut leaves; however, exposure to salinity stress led to severe damages in cytoplasmic membrane, mitochondria, and chloroplast. Notably, *GO priming* alleviated salt-induced plasmolysis and maintained the integrity of thylakoid and plasma membrane (Fig. 7C).

### Graphene oxide increased the peanut productivity

We further investigated the potential role of *GO priming* in peanut productivity under field-grown conditions. *GO priming* significantly increased the peanut pod yield by 12.25% and 13.56% in 2022 and 2023, respectively, compared with non-primed control. For yield related components, no significant difference was observed in plant number per ha; however, *GO priming* significantly increased the 100-pod weight/pod number plant^− 1^ by 5.17/8.16% and 6.38/8.86% in 2022 and 2023, respectively, compared with CK (Table 1).

**Table 1:** Effect of GO on peanut yield and yield related components

Year	Treatment	Pod yield (kg ha^−1 ^)	100-pod weight (g)	Plant number (No. ha^− 1^)	Pod number (No. plant^− 1^)
2022	CK	5334.22 ± 183.54 b	219.40 ± 2.13 b	141,667 ± 3325 a	17.16 ± 0.85 b
	GO	5988.02 ± 265.21 a	230.75 ± 1.56 a	139,815 ± 1667 a	18.56 ± 0.45 a
2023	CK	5342.96 ± 197.36 b	217.44 ± 1.97 b	140,741 ± 1202 a	17.46 ± 0.68 b
	GO	6067.28 ± 188.35 a	231.31 ± 2.33 a	137,963 ± 5774 a	19.00 ± 0.49 a
Source of variance					
Year (Y)		ns	ns	ns	ns
Treatment (T)		***	**	ns	**
Y×T		ns	ns	ns	ns

Data are presented as the means ± standard deviation (SD) of three replications. Different letters in the same column of each year indicate significant differences among treatments (*P* < 0.05). Asterisks indicate significant differences among cultivars (C) and treatments (T). ^**^, ^***^, and ns refer to *P* < 0.01, *P* < 0.001, and non-significant, respectively

## Discussion

In recent years, the profound roles of NMs in plant growth and abiotic stress responses have received a broad spectrum of attention. The utilization of NMs also provides a fruitful avenue for agronomists and farmers to strengthen seedling growth and mitigate crop environmental stress in a more precise and economical way [80–82]. Here we investigate, for the first time, the dominant roles of *GO priming* in promoting seed germination, alleviating seedling salinity stress, and enhancing productivity in peanut plant. The results of the present work may help farmers develop profitable strategies by using NMs like GO in resisting soil salinization.

Literatures advocated that NMs could promote plant growth under both favorable and harsh conditions due to their unique property of high surface-to-volume ratio [83, 84]. In the present work, we were able to visualize the phenotype of GO on the surface of GO-inoculated peanut seeds (Fig. 1C). In conformity with earlier reports, we deduce that the increased surface-to-volume ratio could contribute to the acceleration of nutrient and water assimilation, and consequently increased GR and PSWRB (Fig. 2B). Sugar and protein have been proposed as key components during seed germination [85, 86]. From a metabolomic point of view, GO-primed peanut seeds exhibited increased accumulation of metabolites regarding sugar and protein pathways (Fig. 3). It is, therefore, quite plausible that *GO priming* plays a relevant role in promoting the biosynthesis of seed constituent substances. It is worth mentioning that *GO priming* also induced the secondary metabolomic processes including “Flavonoid biosynthesis” and “Phenylpropanoid biosynthesis” (Figs. 2 and 3). Accumulating evidence validated that flavonoids are a class of natural compounds with nutraceutical and pharmaceutical functions [87, 88], which are also essential for crop abiotic stress responses [89, 90]. In this regard, it appears likely that *GO priming* contributes to establishing a firm defence mechanism via modulating secondary metabolisms in peanut seeds, by which peanut plants could combat the upcoming environmental stress at seedling stage. The above observations prompted us to further elucidate the effects of *GO priming* on peanut salinity resistance at seedling stage.

The Raman spectrum of GO were detected in both stems and roots of peanut seedlings (Fig. 1F and G). Strikingly, dramatic increases in volume, length, and nutrition contents of roots were observed in seedlings of *GO priming* under salinity stress (Figs. 4A, B and D and S4), suggesting that *GO priming* might promote root growth via strengthening the absorption and/or biosynthesis of soil nutrients. From the physiological point of view, *GO priming*-enhanced photosynthesis (Fig. 6D) could contribute to the accumulation of photosynthetic products like FAA under salinity stress (Figs. 5A and S3). On the one hand, the over-accumulation of FAA might participate in the biosynthesis of root structural proteins [91, 92], thus boosting root growth to confer salinity stress (Fig. 4A, B and C). On the other hand, FAA have been long recognized as major components of the osmoregulation system [93, 94], which is responsible for maintaining the cell membrane integrity in root salinity resistance. Combined with transcriptome and metabolomics analysis, more attention have been paid to some enriched pathways associated with phytohormone, carbon, and nitrogen metabolisms in seedlings of *GO priming* under salinity stress. *GO priming* significantly increased the expression of genes like *GLU* and *GLUD1_2* regarding L-Glutamate (L-Glu) biosynthesis, hence promoting the accumulation of L-Glu in peanut roots (Fig. 4E). L-Glu acts as a precursor of environmental stress-related FAA and a long-distance signalling molecule, which is reportedly involved in plant abiotic stress responses [95–97]. Consequently, we deduce that *GO priming* mediated induction of FAA buildup justifies their beneficial roles in promoting root growth, maintaining osmotic pressure and mitigating peanut salinity stress.

Salinity stress impairs the activity of PSII, resulting in loss in the photochemical efficiency [98, 99]. Combined with physiological and transcriptome data, we noticed that *GO priming* dramatically induced some crucial chlorophyll fluorescence parameters such as Fv/Fm, ΦPSII, and ETR (Fig. 6). The induction of ETR can be attributed to the fact that *GO priming* accelerates the electron transport system which was blocked by salinity stress. Meanwhile, *GO priming* protects PSII against over-excitation when the seedlings were suffering from soil salinity, as indicated by the induction of Fv/Fm and ΦPSII [100, 101]. These results were consistent with the TEM observation data, suggesting that salinity-induced loss of integrity of thylakoid membranes has been effectively mitigated by *GO priming* (Fig. 7C). As a result, *GO priming*-enhanced the integrity of thylakoid membranes contributes to the stability of chlorophyll molecules (Fig. 6E), which in turn increases the photosynthetic rate (Fig. 6D).

The multiple functions of phytohormones such as GA and CTK in plant salinity response are becoming increasingly evident. In some cases, the breakdown of GA and CTK resulted in vegetative growth restriction for a better adaptation of the soil salinity [102–104, 99]. Conversely, the excessive accumulation of GA and CTK modulated the chloride exclusion from shoots in response to the harsh environment [105, 106]. Here, data from RNA-seq indicate that the expression of genes involved in the metabolism of growth and stress-related hormones was significantly upregulated under both stress-free and saline conditions after *GO priming*. Specifically, the rate-limiting enzyme KAO and the final step enzyme GA3ox in the GA synthesis pathway were markedly increased under *GO priming* **(**Fig. 4F**)**. Similarly, the key gene *IPT* in the CTK metabolic pathway showed elevated expression levels following *GO priming* when exposed to salinity, which corroborated by our metabolomic results (Fig. 4G). The well-known growth-promoting hormone IAA also exhibited increased expression of the critical *YUCCA* genes under *GO priming*, as shown by RNA-seq data **(**Fig. 4H**)**. These results align with the observed growth and physiological data under various treatments **(**Figs. 4 and 5**)**. Collectively, these observations suggest that *GO priming* enhances plant growth by regulating phytohormones to combat soil salinity. It is worth noting that GO failed to accumulate in peanut leaves (Fig. 1E). Strikingly, outstanding contributions of *GO priming* in protecting the photosystem and enhancing the antioxidant system of peanut leaves have been detected under soil salinity conditions (Figs. 6 and 7). Herein, an interdependently association between the belowground and the aboveground parts of the seedling has been established whereby *GO priming*-induced phytohormones and osmotic regulatory substances might act as signals in maintaining this delicate balance. In summary, the above findings of the current work signified the essentiality of this sophisticated signal transduction mechanisms concerning *GO priming*-induced peanut salinity tolerance, which warrant further experimental evidence.

NMs emerge as promising new materials with immense potential for crop cultivation and breeding. Cost-benefit determinations have revealed that nanofertilizers and nanopesticides contribute significantly to increasing crop revenue while minimizing environmental risks [107, 108]. However, as we integrate these NMs into agricultural practices, it may also address potential environmental health and safety concerns. During the seedling stage (*Experiment II*), although GO was detectable in both roots and stems, it was nearly absent in peanut leaves (Fig. 1E, F and G). This suggests that residual GO enhances peanut salt tolerance, at least partially, through hormonal pathways (Fig. 4). Considering the peanut’s lengthy lifecycle; however, GO accumulation in leaves or pods remains minimal. Further evidence supporting the safety of graphene utilization comes from a study involving ^14^C-labeled graphene in rice, in which ^14^C-labeled graphene reacts with hydroxyl radicals in leaves, leading to its degradation into ^14^CO_2_. Over a 15-day period, graphene accumulation in stems and leaves diminished, with no detectable graphene remaining in rice seeds [109]. Additionally, the polycyclic structure of graphene, akin to lignin and polycyclic aromatic hydrocarbons, renders it susceptible to degradation by lignin peroxidase enzymes secreted by soil microorganisms [110]. Thus, our investigation into the effects of *GO priming* provides a safe strategy for peanut cultivation under both stress-free and salinity conditions.

## Conclusion

**Fig. 8:**
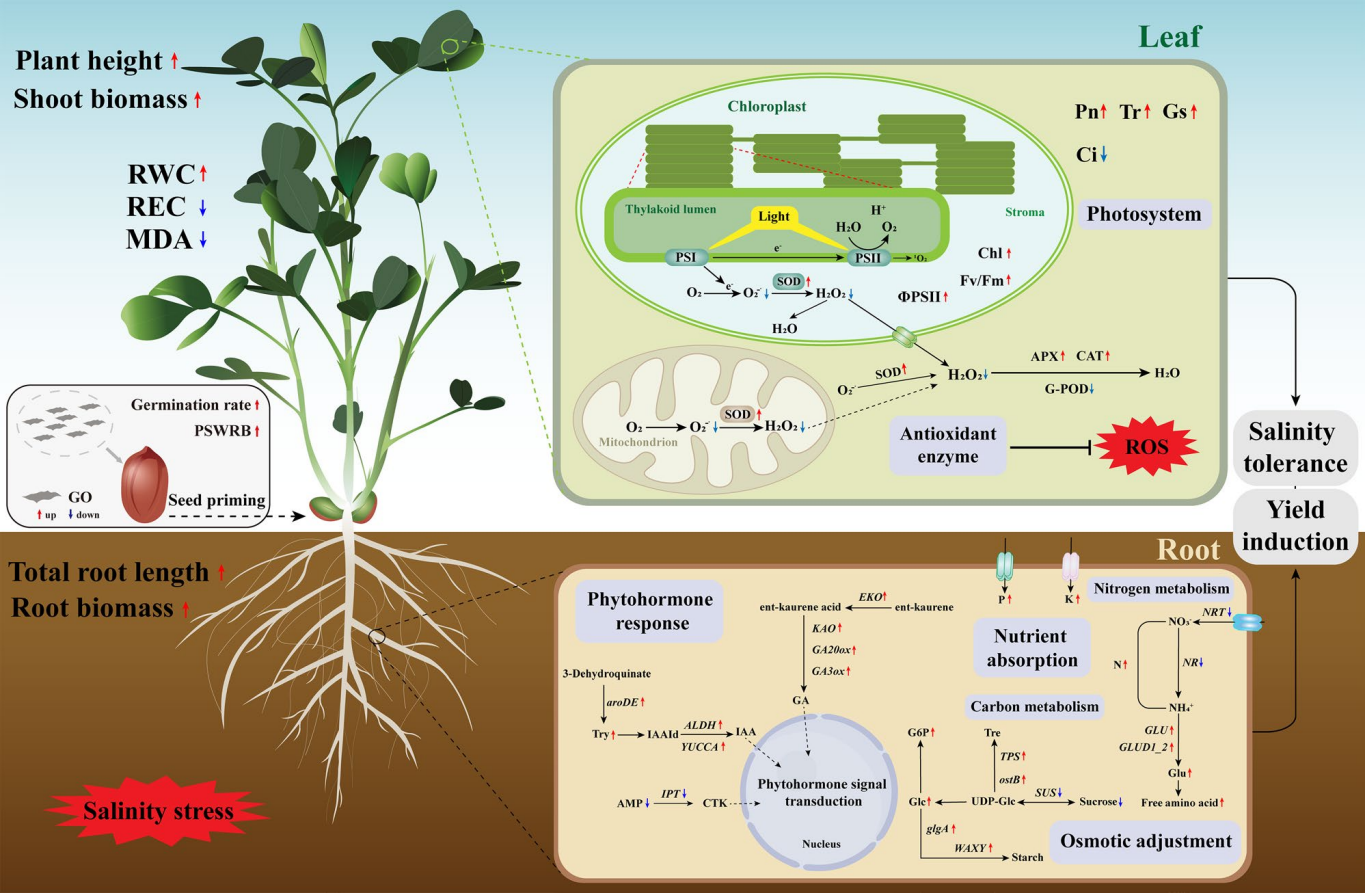
Working model illustrating the mechanisms of seed priming with GO in promoting seed germination and strengthening seedling salinity tolerance of peanut. Red and blue arrows represent up-regulation and down-regulation, respectively

Integrated physiological parameters with transcriptomics and non-target metabolomics, we document that seed priming with 400 mg L^− 1^ GO could increase the seed germination rate and PSWRB of peanut seeds via simulating the biosynthesis of amino acids and secondary metabolites. Furthermore, when the seedlings were exposed to 200 mM NaCl stress, peanut seedlings of *GO priming* exhibited the promotion of plant growth including higher plant height, root length, and plant biomass. In addition, *GO priming* mediated photoprotection of photosynthetic machinery as indicated by the higher Pn, Fv/Fm, ΦPSII, and total chlorophyll content in response to soil salinity. Meanwhile, the activities of antioxidant enzymes including SOD, APX, and CAT were dramatically increased in peanut leaves of *GO priming*, hence reducing salt-induced higher MDA content and REC to maintain plasma membrane integrity. Moreover, *GO priming* also simulated the biosynthesis of some crucial phytohormones (GA, CTK, and IAA) and modulated the metabolisms of carbon and nitrogen in peanut roots, leading to the excessive accumulation of FAA and nutrients in response to salinity stress. Under field-grown conditions, *GO priming* also exhibited higher peanut pod yield with the increased 100-pod weight and pod number per plant (Fig. 8). Nonetheless, the mechanisms concerning *GO priming*-promoted yield formation of peanut pods, especially in late growth stages, should be further elucidated. Moreover, future studies pertaining to the genetic evidence of *GO priming* could provide a more comprehensive understanding of GO-legume interactions.

## Electronic supplementary material

Below is the link to the electronic supplementary material.

Supplementary Material 1

Supplementary Material 2

Supplementary Material 3

Supplementary Material 4

Supplementary Material 5

## Data Availability

The RNA-seq data were deposited to the Sequence Read Archive (SRA) database of the National Center for Biotechnology Information (NCBI, accession number: PRJNA1105759 and PRJNA1105760).
