# Supplementary material for: Seed priming with graphene oxide improves salinity tolerance and increases productivity of peanut through modulating multiple physiological processes
Source: J Nanobiotechnology. 2024 Sep 14;22:565. doi: 10.1186/s12951-024-02832-7 (PMC11401308; doi:10.1186/s12951-024-02832-7)
Supplement: Supplementary file 1 — Supplementary Material 1 [file 12951_2024_2832_MOESM1_ESM.docx]

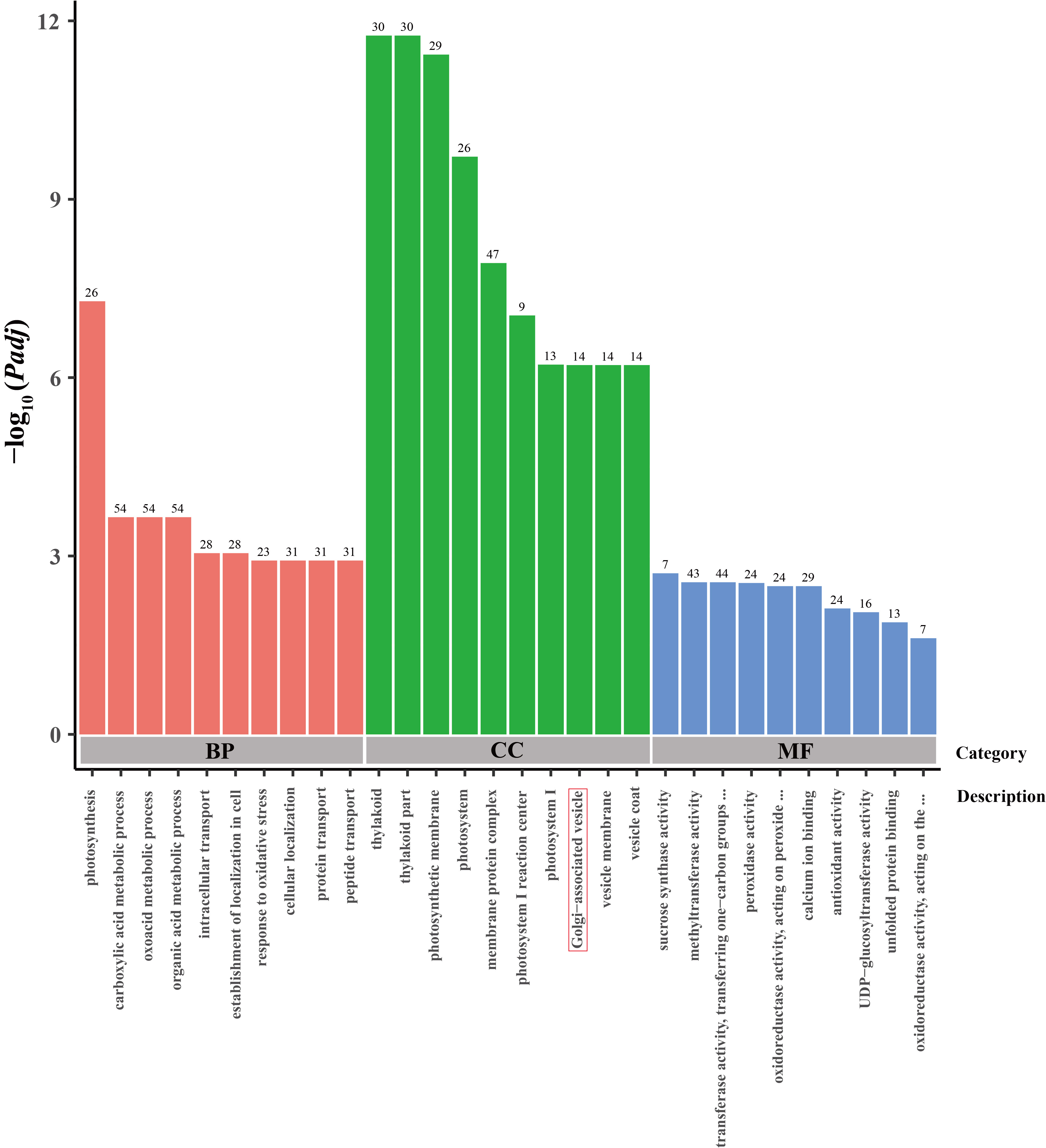
**Fig. S1** Gene Ontology enrichment analysis of GO functional pathways. DEGs were classified into three ontologies, namely biological processes (BPs), molecular functions (MFs), and cellular components (CCs) for the “GO vs CK”. The red, green, and blue colors represent BPs, CCs, and MFs, respectively.





**Fig. S2** Transcriptome analysis of peanut seedlings in different treatments. (**A**) Veen diagram illustrating the number of DEGs identified in “GO vs CK”, “NaCl vs CK” and “GO + NaCl vs CK”. (**B**) Top 20 KEGG enrichment pathways of “GO vs CK”, “NaCl vs CK”, and “GO + NaCl vs CK” were presented by histogram, respectively.





**Fig. S3** Metabolomics analysis of peanut seedlings in different treatments. (**A**) PCA of DAMs in different groups. (**B**) KEGG enrichment pathways. (**C**) Heatmap of metabolite accumulations in different groups.


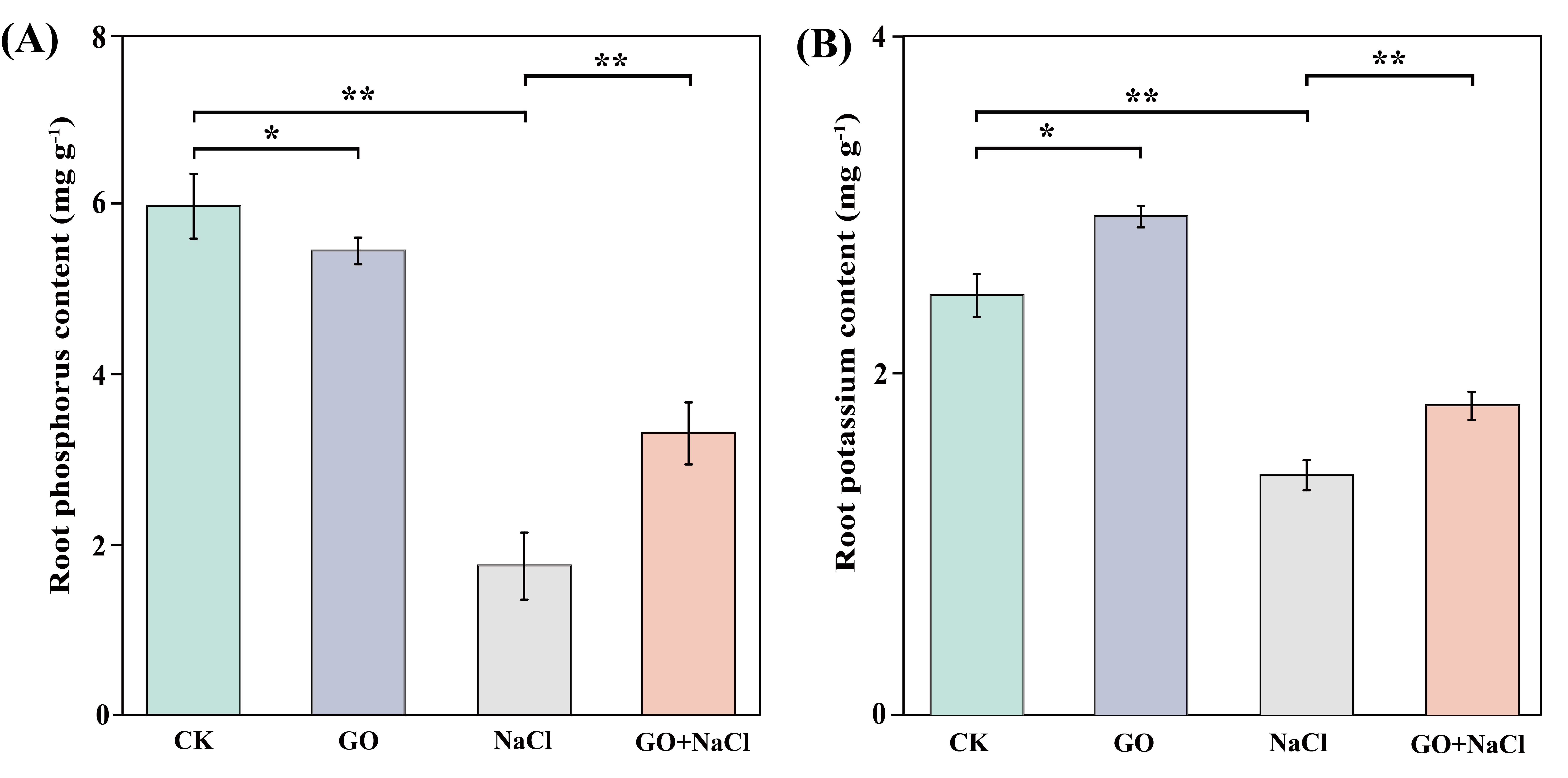


**Fig. S4** Effect of GO on phosphorus content (**A**) and potassium content (**B**) of peanut roots under salinity stress. mean ± SD (n=3), **P*＜0.05, ***P*＜0.01, Tukey's test.


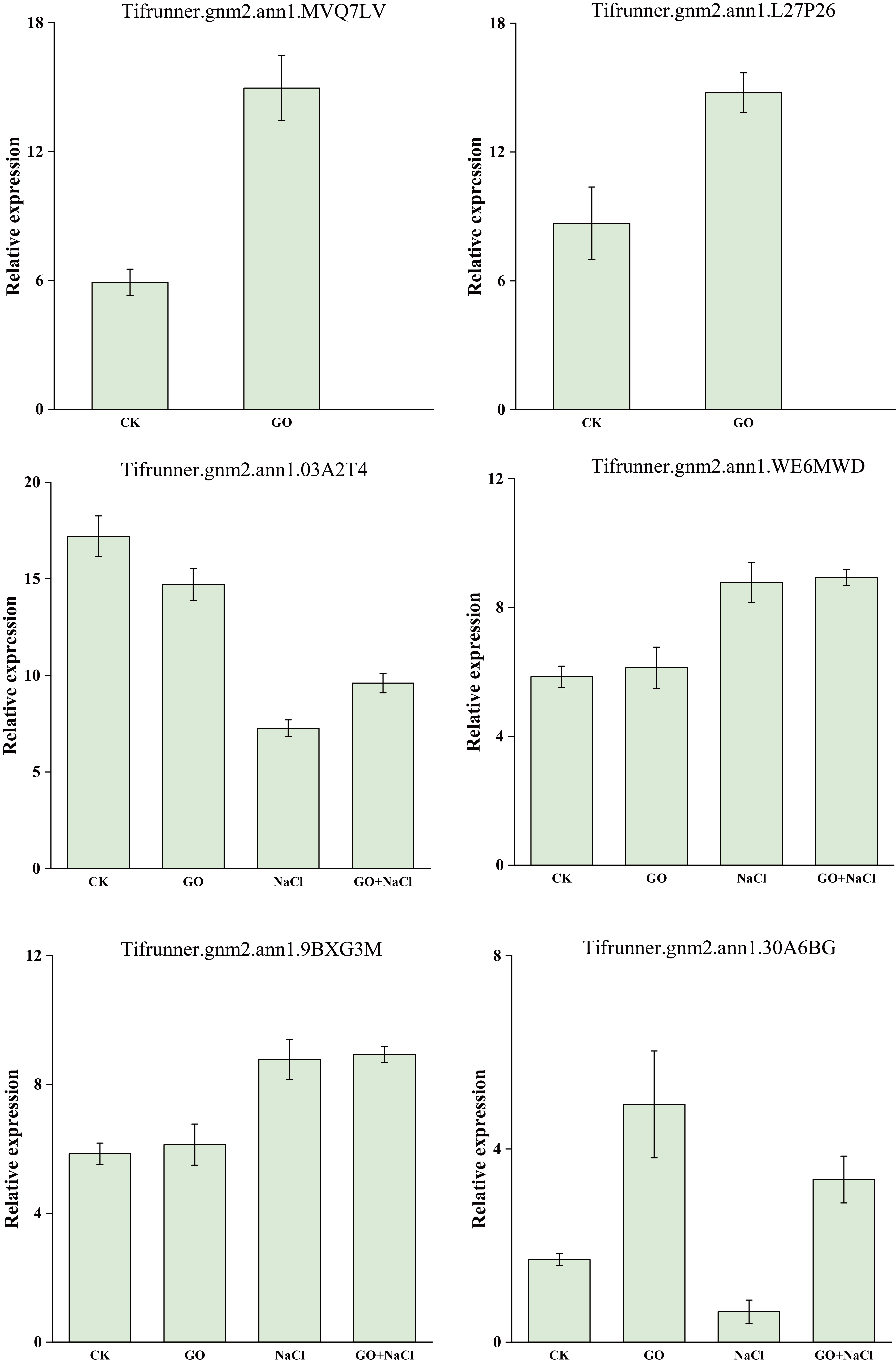


**Fig. S5** qRT-PCR verification of DEGs obtained from RNA-seq results in peanut seeds and seedlings.
