# Supplementary material for: Seed priming with graphene oxide improves salinity tolerance and increases productivity of peanut through modulating multiple physiological processes
Source: J Nanobiotechnology. 2024 Sep 14;22:565. doi: 10.1186/s12951-024-02832-7 (PMC11401308; doi:10.1186/s12951-024-02832-7)
Supplement: Supplementary file 2 — Supplementary Material 2 [file 12951_2024_2832_MOESM2_ESM.docx]

**Table. S1** Physical and chemical properties of the soil in the experimental site.

| **Soil depth** | **Available nitrogen**  **(N) (mg kg^-1^)** | **Available phosphorus**  **(P) (mg kg^-1^)** | **Available potassium**  **(K) (mg kg^-1^)** | **Organic matter**  **(g kg^-1^)** | **pH** |
| --- | --- | --- | --- | --- | --- |
| 0-20 cm | 47.32 | 23.53 | 73.55 | 10.63 | 7.81 |
| 20-40 cm | 28.35 | 16.34 | 56.75 | 6.27 | 7.83 |
