# Supplementary material for: Seed priming with graphene oxide improves salinity tolerance and increases productivity of peanut through modulating multiple physiological processes
Source: J Nanobiotechnology. 2024 Sep 14;22:565. doi: 10.1186/s12951-024-02832-7 (PMC11401308; doi:10.1186/s12951-024-02832-7)
Supplement: Supplementary file 3 — Supplementary Material 3 [file 12951_2024_2832_MOESM3_ESM.docx]

**Table. S2** Primers used in this investigation.

| **Primer ID** | **Sequence** |
| --- | --- |
| Actin-F | TTGGAATGGGTCAGAAGGATGC |
| Actin-R | AGTGGTGCCTCAGTAAGAAGC |
| Tifrunner. gnm2. ann1. L27P26-RT-F | TCCAGGCGTAACCCGAAATC |
| Tifrunner. gnm2. ann1. L27P26-RT-R | CAGCAGTGAGGGGCTTTACA |
| Tifrunner. gnm2. ann1. MVQ7LV -RT-F | CCTTCCAGCATTCCGGCTAT |
| Tifrunner. gnm2. ann1. MVQ7LV -RT-R | ACGTAACCAGCCACAACCAA |
| Tifrunner. gnm2. ann1. 03A2T4-RT-F | TGCAACAGAGGCTGTGCTTA |
| Tifrunner. gnm2. ann1. 03A2T4-RT-R | TGGGATGTCAAGGCCATTGTT |
| Tifrunner. gnm2. ann1. WE6MWD -RT-F | TCTTGGTGGATCTCTGGGGA |
| Tifrunner. gnm2. ann1. WE6MWD -RT-R | TGACAAAGCGTTGGCCTGAT |
| Tifrunner. gnm2. ann1. 30A6BG -RT-F | GTGGTTTCGACGCGAATGTT |
| Tifrunner. gnm2. ann1. 30A6BG -RT-R | CTTCGTCTCCATCGGCAGTC |
| Tifrunner. gnm2. ann1. 9BXG3M -RT-F | AGCTCATCTTCATCGGCTGG |
| Tifrunner. gnm2. ann1. 9BXG3M -RT-R | CCATCCGAGAGGGTCAAACC |
