# Supplementary material for: Seed priming with graphene oxide improves salinity tolerance and increases productivity of peanut through modulating multiple physiological processes
Source: J Nanobiotechnology. 2024 Sep 14;22:565. doi: 10.1186/s12951-024-02832-7 (PMC11401308; doi:10.1186/s12951-024-02832-7)
Supplement: Supplementary file 4 — Supplementary Material 4 [file 12951_2024_2832_MOESM4_ESM.docx]

**Table. S3** Description of the top 19 enriched KEGG metabolic pathways in GO-treated peanut seeds.

| **KEGGID** | **Description** | ***P-value*** | ***Padj*** | **All** | **Up** | **Down** |
| --- | --- | --- | --- | --- | --- | --- |
| gmx00195 | Photosynthesis | 3.58E-12 | 4.33E-10 | 32 | 31 | 1 |
| gmx00196 | Photosynthesis - antenna proteins | 1.27E-07 | 7.67E-06 | 12 | 12 | 0 |
| gmx04141 | Protein processing in endoplasmic reticulum | 1.18E-06 | 4.77E-05 | 53 | 8 | 45 |
| gmx00520 | Amino sugar and nucleotide sugar metabolism | 0.000198 | 0.005979 | 35 | 12 | 23 |
| gmx00260 | Glycine, serine and threonine metabolism | 0.000341 | 0.008263 | 22 | 4 | 18 |
| gmx00940 | Phenylpropanoid biosynthesis | 0.001297 | 0.026151 | 30 | 3 | 27 |
| gmx00941 | Flavonoid biosynthesis | 0.003942 | 0.068143 | 18 | 2 | 16 |
| gmx00650 | Butanoate metabolism | 0.004579 | 0.069251 | 10 | 4 | 6 |
| gmx00960 | Tropane, piperidine and pyridine alkaloid biosynthesis | 0.008105 | 0.108966 | 16 | 0 | 16 |
| gmx00020 | Citrate cycle (TCA cycle) | 0.014366 | 0.173835 | 16 | 4 | 12 |
| gmx01250 | Biosynthesis of nucleotide sugars | 0.016309 | 0.179396 | 22 | 6 | 16 |
| gmx00500 | Starch and sucrose metabolism | 0.019014 | 0.191721 | 34 | 13 | 21 |
| gmx00510 | N-Glycan biosynthesis | 0.025352 | 0.209612 | 12 | 3 | 9 |
| gmx00970 | Aminoacyl-tRNA biosynthesis | 0.026056 | 0.209612 | 15 | 5 | 10 |
| gmx00450 | Seleno compound metabolism | 0.02651 | 0.209612 | 6 | 0 | 6 |
| gmx04145 | Phagosome | 0.028045 | 0.209612 | 19 | 5 | 14 |
| gmx00010 | Glycolysis / Gluconeogenesis | 0.029502 | 0.209612 | 31 | 7 | 24 |
| gmx00280 | Valine, leucine and isoleucine degradation | 0.031182 | 0.209612 | 15 | 8 | 7 |
| gmx00290 | Valine, leucine and isoleucine biosynthesis | 0.033955 | 0.216237 | 6 | 2 | 4 |
